# Magnetic resonance imaging radiomics-based prediction of clinically significant prostate cancer in equivocal PI-RADS 3 lesions in the transitional zone

**DOI:** 10.3389/fonc.2023.1247682

**Published:** 2023-11-23

**Authors:** Ying-Ying Zhao, Mei-Lian Xiong, Yue-Feng Liu, Li-Juan Duan, Jia-Li Chen, Zhen Xing, Yan-Shun Lin, Tan-Hui Chen

**Affiliations:** ^1^ Department of Radiology, Fuqing City Hospital Affiliated to Fujian Medical University, Fuqing, China; ^2^ Department of Radiology, The First Affiliated Hospital of Fujian Medical University, Fuzhou, China; ^3^ Department of Ophthalmology Surgery, Taihe Hospital, Hubei University of Medicine, Shiyan, China

**Keywords:** magnetic resonance imaging, prostate cancer, PI-RADS, transitional zone, radiomics

## Abstract

**Purpose:**

This bi-institutional study aimed to establish a robust model for predicting clinically significant prostate cancer (csPCa) (pathological grade group ≥ 2) in PI-RADS 3 lesions in the transition zone by comparing the performance of combination models.

**Materials and methods:**

This study included 243 consecutive men who underwent 3-Tesla magnetic resonance imaging (MRI) and ultrasound-guided transrectal biopsy from January 2020 and April 2022 which is divided into a training cohort of 170 patients and a separate testing cohort of 73 patients. T2WI and DWI images were manually segmented for PI-RADS 3 lesions for the mean ADC and radiomic analysis. Predictive clinical factors were identified using both univariate and multivariate logistic models. The least absolute shrinkage and selection operator (LASSO) regression models were deployed for feature selection and for constructing radiomic signatures. We developed nine models utilizing clinical factors, radiological features, and radiomics, leveraging logistic and XGboost methods. The performances of these models was subsequently compared using Receiver Operating Characteristic (ROC) analysis and the Delong test.

**Results:**

Out of the 243 participants with a median age of 70 years, 30 were diagnosed with csPCa, leaving 213 without a csPCa diagnosis. Prostate-specific antigen density (PSAD) stood out as the only significant clinical factor (odds ratio [OR], 1.068; 95% confidence interval [CI], 1.029–1.115), discovered through the univariate and multivariate logistic models. Seven radiomic features correlated with csPCa prediction. Notably, the XGboost model outperformed eight other models (AUC of the training cohort: 0.949, and validation cohort: 0.913). However, it did not surpass the PSAD+MADC model (P > 0.05) in the training and testing cohorts (AUC, 0.949 vs. 0.888 and 0.913 vs. 0.854, respectively).

**Conclusion:**

The machine learning XGboost model presented the best performance in predicting csPCa in PI-RADS 3 lesions within the transitional zone. However, the addition of radiomic classifiers did not display any significant enhancement over the compound model of clinical and radiological findings. The most exemplary and generalized option for quantitative prostate evaluation was Mean ADC+PSAD.

## 1 Introduction

Prostate cancer is the most common cancer affecting men worldwide (1), with a lifetime risk as high as 37% (2). By 2040, global prostate cancer incidences are projected to rise to nearly 2.3 million new cases and 740 000 deaths (3). Multi-Magnetic resonance imaging (mpMRI) of the prostate, which helps improve the detection, localization, and staging of prostate cancer (PCa), has been established as the de facto standard for the imaging assessment of suspected PCa (4). A large-sample study demonstrated that utilizing MRI for initial screening before biopsy can minimize needless biopsies by approximately half for cases with a PI-RADS score of 3 or higher, and prevent the overdiagnosis of clinically insignificant lesions (5).

The Prostate Imaging Reporting and Data System (PI-RADS) has undergone continuous refinement and updates since its conception, allowing for more standardized assessments of prostate lesions. The most recent iteration, PI-RADSv2.1 revised in 2019, standardizes the terminology, interpretation, and contents of MRI reports (6). Lesions are classified into five categories (1 to 5), based on their anatomical location and MRI signal changes. Higher categories correlate with a higher probability of detecting clinically significant prostate cancer (csPCa). Lesions categorized as PI-RADS 1 and 2 bear an exceedingly low cancer detection rate (CDR) of less than 5% (7, 8) and necessitate only follow-up. In contrast, lesions classified as PI-RADS 4 and 5 have an extremely high CDR (40–80%) (7, 8) calling for further biopsy. However, PI-RADS 3 lesions present a moderate CDR, indicating an ambiguous risk of malignancy.

PI-RADS 3 lesions are frequently identified in patients undergoing MRI examinations, with reported incidences ranging from 22%–32% (9); However, most studies indicate a relatively low detection rate for csPCa, between 2%–22.9% (10, 11). Concurrently, the false negative rate for csPCa is notably high at 16.2% for cases with PI-RADS scores of 3 or higher (5). Current guidelines offer no explicit direction for subsequent treatment of PI-RADS 3 lesions, thereby presenting a dilemma for urologists in deciding between follow-up prostate-specific antigen (PSA) testing and imaging monitoring, or immediate biopsy. It is crucial to selectively submit patients likely to have csPCa to undergo prostate biopsy, maximizing the benefits from the procedure and potential aggressive treatment strategies.

Although the implementation of PI-RADSv2.1 has boosted the precision in identifying csPCa, PI-RADS 3 lesions remain elusive within the “gray zone” of mpMRI evaluations, especially for the transition zone (TZ). Benign prostatic hyperplasia, a common condition in elderly men, creates a degree of organized chaos within the TZ, hampering accurate lesion categorization (12). Recent studies suggest exploiting radiomic features and mean apparent diffusion coefficient (ADC) values to quantitively evaluate MRI enhances diagnostic accuracy for TZ lesions over mere qualitative PI-RADS assessment (13–15). Engel et al. reported that the risk stratification for prostatic TZ lesions could be improved through a quantitative diffusion-weighted imaging (DWI) analysis (4). Another study demonstrated an achievable specificity and sensitivity through downgrading PI-RADS lesions at or above 4 based on mean ADC values or machine learning algorithms (15). Ultimately, radiomics hold potential in algorithmically identifying csPCa in PI-RADS 3 lesions (13–16). A greater balance between biopsy-associated complications, overdiagnosis, and overlooking csPCa diagnosis might be achieved using radiomics prostate MRI. For patients with both PI-RADS 3 and a low risk of csPCa, immediate biopsy can possibly be deferred. However, previous studies of this nature typically involved smaller cohorts from a single institution and lacked distinction between PZ and TZ lesions (17).

Therefore, we hypothesized that the characteristics of detected TZ lesions can be improved through radiomics. This study aimed to assess different algorithm models for risk stratification among patients with PI-RADS 3 in the TZ, using a combination of individual clinical characteristics and radiological data.

## 2 Materials and methods

### 2.1 Demographic information and clinical data

**Figure 1:**
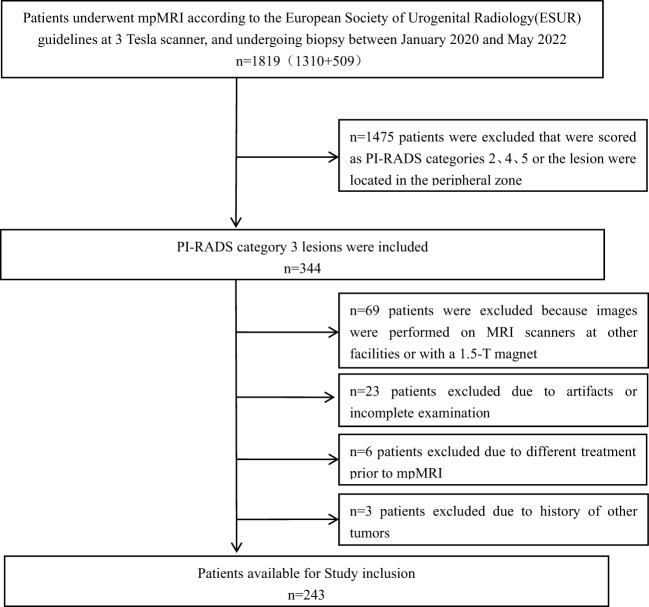
Flowchart of the study population.

This retrospective study included patients from two institutions (The First Affiliated Hospital of Fujian Medical University and Fuqing Hospital). The institutional ethics committee approved this study and waived the requirement for informed consent. Data were retrospectively collected from men who underwent MRI and biopsy examinations between January 2020 and April 2022 according to the following eligibility criteria: (a) men with PI-RADS 3 lesions (v2.1 standard), (b) PI-RADS 3 lesions confirmed by pathology and matched to the MR images in the same region, and (c) ultrasound-guided prostate biopsy or radical surgery performed within 1 month of the MRI examination. The exclusion criteria were: (a) biopsy or a history of treatment (antihormonal therapy, radiation therapy, focal therapy, or prostatectomy) for prostate cancer prior to the MRI examination, (b) multiple primary cancers or a previous history of cancer, (c) incomplete sequences or severe artefacts on MRI images, and (d) any PI-RADS 4 or 5 lesions. **Figure 1** presents the flowchart of the inclusion and exclusion criteria of the study.

The following clinical and laboratory data were collected: age, the most recent serological value of total prostate-specific antigen (tPSA; ng/mL), free prostate-specific antigen (fPSA; ng/mL), fPSA/tPSA (f/t), prostate volume(V), PSA density (total PSA/prostatic volume ratio [PSAD]) during MRI examination and final histopathological analysis, and mean ADC value (mm^2^/s). The mean ADC value was calculated in volumes of interest (VOIs), encompassing the entire lesion without exceeding the lesion margins. **Table 1** presents the baseline epidemiologic and clinical characteristics, including tumor location, pathological findings, and clinical assessment.

**Table 1:** The Characteristics of Demographic and Clinical Data of Patients with PIRADs 3 Lesions on MRI.

	level	Overall	No csPCa	csPCa	*p*
n		(n=243)	(n=213)	(n=30)	
GGG (%)	0	183 (75.3)	183 (85.9)	0 (0.0)	<0.001
	1	30 (12.3)	30 (14.1)	0 (0.0)	
	2	19 (7.8)	0 (0.0)	19 (63.3)	
	3	8 (3.3)	0 (0.0)	8 (26.7)	
	4	3 (1.2)	0 (0.0)	3 (10.0)	
age (median [IQR])		70	70	72	0.026
		[66.00, 75.00]	[65.00, 74.00]	[68.00, 76.00]	
MADC (median [IQR]×10-4)		8.22	8.39	6.98	<0.001
		[7.51, 8.83]	[7.68, 8.91]	[6.43, 7.75]
MT2WI (median [IQR]×102)		2.85	2.85	2.81	0.742
		[2.52, 3.38]	[2.54, 3.38]	[2.42, 3.53]
RT2WI (median [IQR])		3.05	3.06	2.98	0.346
		[2.66, 3.40]	[2.66, 3.43]	[2.75, 3.18]
V (median [IQR])		50	51.94	43.04	0.012
		[35.19, 66.60]	[35.70, 69.18]	[23,68, 52.56]
PSAD (median [IQR]×10-2)		17.69	16.54	38.34	<0.001
		[10.10, 29.32]	[9.81, 25.38]	[19.19, 53.07]
tPSA (median [IQR])		8.65	8.38	10.67	0.009
		[5.23, 13.55]	[5.07, 12.30]	[8.17, 19.18]
fPSA (median [IQR])		1.45	1.42	1.94	0.029
		[0.95, 2.20]	[0.90, 2.14]	[1.35, 2.78]
f/t (median [IQR])		0.17	0.17	0.13	0.044
		[0.12, 0.22]	[0.14, 0.22]	[0.10, 0.20]

GGG, Gleason grade group; MADC,mean apparent diffusion coefficient; V, the volume of prostate; PSAD, prostate specific antigen density; tPSA, total prostate specific antigen; fPSA,free prostate specific antigen; f/t,fPSA/tPSA.

A 12-core systematic biopsy was performed by urologists with three to five years of transrectal ultrasound-guided prostate biopsy experience. Based on biopsy results, the patients were divided into two cohorts: the csPCa and no csPCa (benign and Grade Group 1 [GG1]) groups. The primary endpoint of csPCa was defined as patients with≥GG2 (Gleason 3 + 4) prostate cancer.

### 2.2 MRI examination

During the study period, prostate MRI was performed at Institution 1 using a 3.0T scanner (Spectra; Siemens Healthineers), whereas it was performed using a 3.0T MRI system (Philips Ingenia, Amsterdam, the Netherlands) at Institution 2. Standard multichannel body coils and integrated spine phased-array coils were used according to the guidelines of the European Society of Urogenital Radiology (18). **Appendix Table 1** summarizes the details of the MRI protocols of each institution.

**Appendix 1:** Acquisition Parameters of the Multiparametric MRI Protocol for both institution.

Parameter	T2WI		DWI	
	Institution 1	Institution 2	Institution 1	Institution 2
Echo time (msec)	72	93	72	93
Repetition time (msec)	4000	5960	4000	6900
Flip angle (°)	90	150	–	–
Matrix	128×128	256×256	128×128	256×256
Field of view (mm2)	360×360	160×160	360×360	190×260
Number of slices	36	40	32	40
Slice thickness (mm)	4	3	4	3
spacing between slices	0.5	0	1	0
b-values (s/mm2)	–	–	100/800/2000	50/800/1500

### 2.3 MRI lesion segmentation

To confirm that the lesions were classified as PI-RADS 3 as per the PI-RADS v2.1 guidelines, the MR images were interpreted by two radiologists (Y. Y. Z. and M. L. X.) with 6 and 10 years of experience in prostate MRI interpretation, respectively, who were blinded to the pathological data. In cases of disagreement, a final consensus was reached by re-reading.

Axial T2-weighted and diffusion-weighted images in DICOM format were downloaded from the picture archiving and communication system (PACS). The MRI index lesions were manually segmented by an investigator (Y. Y. Z.). Given the importance of heterogeneity analysis while avoiding partial volume effects, VOIs encompassing the entire lesion, including bleeding, necrosis, and cystic areas, the urethra, ejaculatory duct, and other normal anatomical structures were drawn on each slice with the lesion. Segmentation was performed under the supervision of another radiologist (T.H.C. with 20 years of experience in prostate MRI), using the dedicated software ITK-SNAP (version 3.8.0 for Win, http://www.itksnap.org/). In addition, segmentation was also performed separately on axial T2-weighted and ADC images. The background obturator internus in the corresponding or adjacent layers was segmented for reference, excluding the muscle steatosis area while encompassing at least 50 voxels in at least three adjacent sections.

For the intraobserver and interobserver agreement evaluation in manual segmentation, we randomly selected 50 patients, and their ROIs were delineated 1 month later by the same radiologists (Y. Y. Z. and M. L. X.).

### 2.4 Image postprocessing and analysis

**Figure 2:**
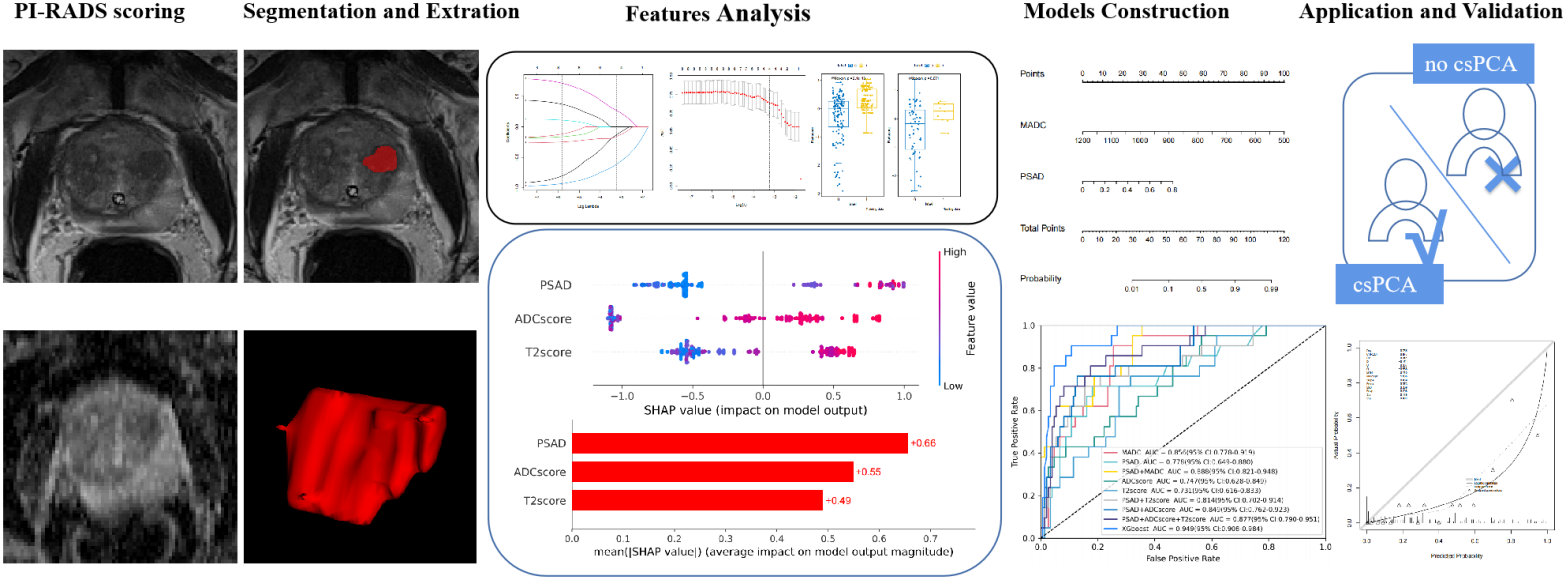
Radiomics analysis workflow. Radiomics features were extracted from both T2-weighted images (T2WI) and apparent diffusion coefficient (ADC) maps. The Student’s *t*-test or Mann–Whitney U-test and the least absolute shrinkage selection operator were used for feature selection, and the models were constructed based on logistic regression and XGboost methods for predicting clinically significant prostate cancer.

T2-weighted images were normalized by dividing the voxel intensities by the mean value of the background obturator internus tissue. Since ADC is a quantitative measurement, it was not normalized. Radiomic feature calculations were performed using the pyradiomics package of Python 3.7.1. (https://github.com/Radiomics/pyradiomics) (18) according to the analytical steps depicted in **Figure 2**. Within each VOI, 14 volume and shape features, 198 first-order histogram features, 264 grey-level co-occurrence matrix (GLCM) features, 154 grey-level dependence matrix (GLDM) features, 176 grey-level run length matrix (GLRLM) features, 176 grey-level size zone matrix (GLSZM) features, and 55 neighboring grey tone difference matrix (NGTDM) features were calculated, resulting in 1037 features per VOI. These features were calculated on both the ADC maps and T2-weighted images; thus, a total of 2074 radiomics features were obtained for each lesion.

### 2.5 Radiomics feature selection and signature building

All patients were randomly stratified into the training and testing cohorts in a 7:3 ratio. The mean ADC was extracted from the radiomic dataset for separate analyses. Missing data were analyzed using the Random Forest Multiple Interpolation method (R language mice package). The features with ICC < 0.75 were filtered out. Subsequently, upsampling was used to address sample imbalance in the training cohort, and the Student’s *t*-test or Mann–Whitney U-test was used for preliminary feature selection, which was determined using the Shapiro–Wilk and Levene’s tests. The least absolute shrinkage selection operator (LASSO)-logistic regression model was used to select the predictive features, and the radiomics signature (rad score) was calculated by adding the selected radiomics features, weighted by their respective coefficients. This procedure was performed separately on the T2-weighted and ADC images.

### 2.6 Creation and verification of model

Multivariate logistic regression analysis was used to identify independent predictive clinical factors. Prediction models were established based on clinical variables (clinical model), radiological features (radiological model), radiomic signatures (radiomics Model), and a combination of clinical variables, radiological features, and radiomics features (logistic regression and XGboost models) to generate a quantitative predictive tool for csPCa diagnosis. Calibration curves were used to evaluate the robustness of the model. Decision curve analysis (DCA) was used to evaluate the net benefit of the model for clinical decision-making at different threshold probabilities.

### 2.7 Statistical analysis

For demographic data, continuous variables were analyzed using Student’s *t*-test or Mann–Whitney U test, as determined by Shapiro–Wilk and Levene’s test. Continuous variables with normal distribution were presented as mean ± standard deviation. Continuous variables with non-normal distribution were presented as median (inter-quartile range [IQR]). Categorical variables were analyzed using the chi-square test or Fisher’s exact test. Univariate and multivariate logistic regression analyses were used to identify the significant predictors of csPCa. LASSO logistic regression analysis was used for screening the predictive radiomics features. The eXtreme Gradient Boosting (XGboost) model was created with stratified 10-fold cross-validation, and a grid search was performed to identify the optimal hyperparameters for training using the GridSearchCV function in Scikit-learn (estimated by ten-fold cross-validation). The diagnostic performance of different models for the prediction of csPCa was assessed using receiver operating characteristic (ROC) curve analysis and by calculating the accuracy, sensitivity, specificity, and area under the ROC curve (AUC) with 95% CI. The Delong test was used to compare the performance of the different models, regardless of whether they differed significantly. All data analyses were performed using Python (version 4.0.1; https://www.r-project.org) and R (version 3.7.3; https://www.python.org/downloads/) software. All tests were two-sided, with statistical significance set at P ≤ 0.05.

## 3 Results

### 3.1 Demographic information and clinical data

This study included 243 patients (median age, 70 years; IQR, 66–75 years). Prostate biopsy revealed that 213 (87.6%) patients did not have csPCa [183(75.3%) men had no cancer, and 30 (12.3%) had GG1], and 30 (12.3%) patients had csPCa. The detection rate for csPCa was equal to 13.7% (23/168 cases) vs 9.3% (7/75 cases) with PI-RADS 3 lesions diagnosed in the institution 1 vs the institution 2. The median PSA level was 8.65 ng/ml, with a mean prostate volume was 50.0 mL, and the median PSAD was 0.17 ng/mL^2^. The patients were randomly allocated to the training (N = 170) and testing (N = 73) cohorts. **Table 1** presents the results of the comparison between the clinical factors of the csPCa and no csPCa groups.

### 3.2 Radiomics features selection and signature building

**Figure 3:**
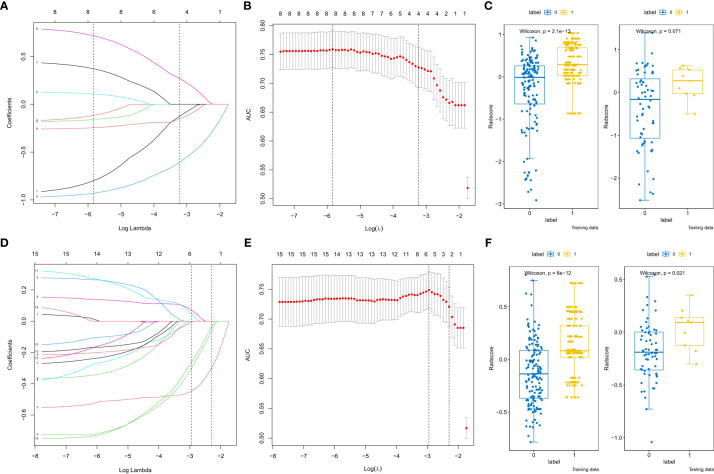
Radiomics features selection by the least absolute shrinkage selection operator. **(A, D)** Coefficient profiles of radiomics features of the apparent diffusion coefficient (ADC) and T2-weighted images (T2WI). **(B, E)** The adjustment penalty parameter λ is -3.229 ×10^-4^ and -2.294×10^-4^ for the ADC and T2WI, and seven features were selected according to 10-fold cross-validation. **(C, F)** The boxplot of radscores of the csPCa and No csPCa groups in the training and testing cohorts of the ADC and T2WI.

Initially, 2074 features were generated from the original T2-weighted and ADC imaging data, and 233 features related to csPCa diagnosis were selected. Highly correlated features were discarded (correlation between two variables > 0.6). Subsequently, the seven most predictive features were selected from the T2-weighted and ADC images using LASSO-logistic regression (**Figure 3** and **Table 2**). The radiomic signature was then calculated by weighting their respective coefficients. The boxplot depicted in **Figure 3** presents the differences between the two groups.

**Table 2:** The final 7 radiomics features selected from T2WI and ADC.

Features	Coef	OR
ADC.wavelet.HLH_glszm_SizeZoneNonUniformityNormalized	-0.60887	0.543965
ADC.wavelet.LLL_firstorder_10Percentile	-0.15362	0.857601
ADC.exponential_firstorder_Energy	-0.12684	0.880874
ADC.wavelet.HHL_glrlm_LongRunHighGrayLevelEmphasis	0.270977	1.311245
T2WI.original_shape_Sphericity	-0.29877	0.741728
T2WI.wavelet.LHL_glcm_MCC	-0.06151	0.940344
T2WI.wavelet.LLL_gldm_LargeDependenceLowGrayLevelEmphasis	-0.03154	0.968952

### 3.3 Development and validation of individualized logistic prediction models

Univariable logistic regression analysis of all potential factors identified age (odds ratio [OR], 1.075; 95% confidence interval [CI], 1.006–1.153), V (OR, 0.987; 95% CI, 0.974–0.998), PSAD (OR, 1.07; 95% CI, 1.034–1.113), and MADC (OR, 0.242; 95% CI, 0.126–0.422) as the independent parameters for csPCa prediction. When age, V, PSAD, and MADC were included in the multivariate logistic regression analysis, only PSAD and MADC remained significantly correlated with tumor diagnosis (**Table 3**). **Table 4** presents the eight models built for predicting csPCa using radiomics, a clinical variable, and the MADC values.

**Table 3:** Results of univariate and multivariate logistic regression analyses.

Facotrs	Uni_OR	Uni_95%CI	Uni_P	Mul_OR	Mul_95%CI	Mul_*P*
age	1.075	(1.006~1.153)	0.036	1.067	(0.968~1.178)	0.188
T	1.034	(0.99~1.076)	0.117	–	–	–
F	1.276	(0.969~1.654)	0.07	–	–	–
F/T	0.174	(0~1.228)	0.588	–	–	–
V	0.987	(0.974~0.998)	0.035	0.995	(0.981~1.008)	0.506
PSAD	1.07	(1.034~1.113)	<0.001	1.068	(1.029~1.115)	0.001
MT2WI	0.902	(0.525~1.373)	0.666	–	–	–
RT2WI	0.854	(0.356~1.96)	0.715	–	–	–
MADC	0.242	(0.126~0.422)	<0.001	0.231	(0.107~0.437)	<0.001

**Table 4:** The performance of different models in training and testing cohorts for predicting tumor diagnosis in csPCa patients.

	Model	AUC (95% CI)	Accuracy	Sensitivity	Specificity
**Training**	MADC	0.856 (0.782-0.923)	0.765	0.905	0.745
	PSAD	0.778 (0.661-0.888)	0.806	0.714	0.819
	ADCscore	0.747 (0.640-0.850)	0.5	0.952	0.436
	T2score	0.731 (0.616-0.838)	0.694	0.762	0.685
	PSAD+MADC	0.888 (0.814-0.943)	0.688	1	0.644
	PSAD+T2score	0.814 (0.689-0.913)	0.829	0.714	0.846
	PSAD+ADCscore	0.849 (0.752-0.929)	0.859	0.762	0.872
	PSAD+ADCscore+T2score	0.877 (0.791-0.954)	0.824	0.81	0.826
	XGboost	0.949 (0.904-0.983)	0.894	0.905	0.893
**Testing**	MADC	0.788 (0.628-0.920)	0.699	0.778	0.688
	PSAD	0.620 (0.371-0.846)	0.795	0.556	0.828
	ADCscore	0.688 (0.540-0.818)	0.548	0.889	0.5
	T2score	0.740 (0.557-0.882)	0.767	0.556	0.797
	PSAD+MADC	0.854 (0.733-0.952)	0.63	0.889	0.594
	PSAD+T2score	0.809 (0.676-0.922)	0.781	0.556	0.812
	PSAD+ADCscore	0.757 (0.627-0.877)	0.74	0.333	0.797
	PSAD+ADCscore+T2score	0.812 (0.684-0.920)	0.822	0.667	0.844
	XGboost	0.913 (0.816-0.984)	0.904	0.889	0.906

MADC, mean ADC; PSAD, PSA density; XGboost, XGboost machine learning model.

### 3.4 Development of the XGboost prediction model

**Figure 4:**
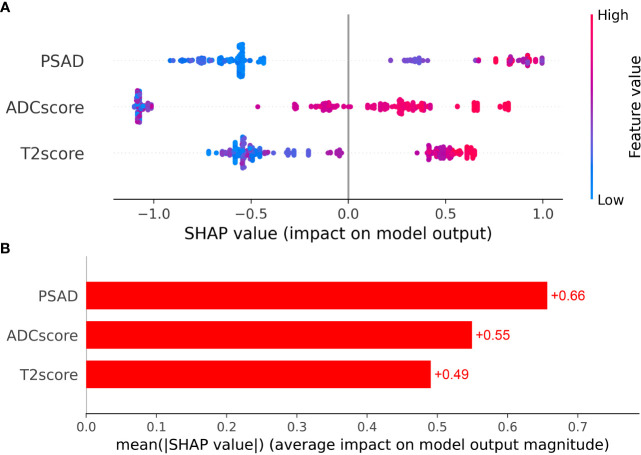
**(A)** The Beeswarm plot depicts the predictive value of each feature for each patient, and **(B)** the bar plot depicts the importance of each feature.

Clinical factors (PSAD) and radiomic signatures were identified as the predictors most significantly associated with csPCa diagnosis. Therefore, these three features were employed as the input variables, whereas diagnostic efficiency was considered the output variance. The XGboost model hyperparameters were optimized using grid search and ten-fold cross-validation. The other parameters were set to default values. The detailed weights of the trained XGboost with the PSAD, T2 score, and ADC score for predicting csPCa are presented in **Figure 4**.

### 3.5 Performance comparisons of models

**Figure 5:**
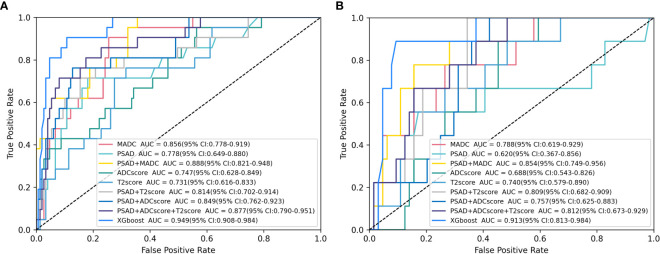
Receiver operating characteristic (ROC) analysis of the parameter models in clinically significant prostate cancer (csPCa) of PI-RADS 3 Lesions prediction. **(A, B)** ROC curves for selected logistic models and XGboost models tested for csPCa prediction in the **(A)** training cohort and **(B)** testing cohort. Plus signs indicate parameter combinations in the multivariable models, and data in brackets are 95% confidence intervals. See **Table 4** for corresponding area under the ROC curve (AUC) values and additional models.

As shown in **Tables 4**, **5**, and **Figure 5**, MADC was found to be the best-performing single-parameter model, with an AUC of 0.856 (95% CI, 0.782–0.923), and 0.788 (95% CI, 0.628–0.920) in the training and testing cohorts, respectively (**Figure 4** and **Table 4**). The best combined models were PSAD + MADC (AUC, 0.888 [95% CI, 0.814–0.943], and 0.854 [95% CI, 0.733–0.952] in the training and testing cohorts, respectively) and PSAD + ADC score + T2 score (0.877 [95% CI, 0.791–0.954], and 0.812 [95% CI, 0.684–0.920] in the training and testing cohorts, respectively) (**Figure 5** and **Table 4**). However, they showed no evidence of improvement compared with the MADC model (*P* =0.162 and *P* = 0.687 in the training cohorts, respectively, and P =0.303 and P = 0.818 in the testing cohorts, respectively).

**Table 5:** The performance comparisons of different models in training and testing cohorts.

Model	MADC	PSAD	PSAD + MADC	ADCscore	T2score	PSAD + T2score	PSAD + ADCscore	PSAD + ADCscore + T2score	XGboost
**MADC**	/	0.219	0.162	0.099	0.046	0.505	0.909	0.687	0.023
**PSAD**	0.322	/	0.014	0.674	0.467	0.247	0.019	0.005	0.000
**PSAD + MADC**	0.303	0.06	/	0.031	0.007	0.132	0.357	0.805	0.075
**ADCscore**	0.367	0.684	0.078	/	0.857	0.38	0.047	0.018	0.000
**T2score**	0.653	0.519	0.295	0.534	/	0.049	0.061	0.002	0.000
**PSAD + T2score**	0.846	0.043	0.513	0.19	0.493	/	0.377	0.022	0.001
**PSAD + ADCscore**	0.771	0.183	0.146	0.337	0.88	0.304	/	0.18	0.004
**PSAD + ADCscore**	0.818	0.158	0.611	0.017	0.34	0.953	0.307	/	0.003
**+ T2score**									
XGboost	0.133	0.038	0.368	0.000	0.008	0.098	0.015	0.018	/

Based on Delong.test, the upper right of the diagonal (yellow) was the P value of model comparisons in the training set, and the down left of the diagonal (blue) was the P value of model comparisons in the testing set.

The XGboost model demonstrated the highest performance for predicting csPCa, with an AUC of 0.949 (95% CI, 0.904–0.983) and 0.913 (95% CI, 0.816–0.984) in the training and testing cohorts, respectively. Significant differences were observed between the AUCs of the XGboost model and the other five models (PSAD, ADC score, T2 score, PSAD + ADC score, and PSAD + ADC score + T2 score) (**Figure 4** and **Tables 4**, **5**). The AUCs of the training cohort were 0.949 vs. 0.778 vs. 0.747 vs. 0.731 vs. 0.849 vs. 0.877. The AUCs of the validation cohort were 0.913 vs. 0.620 vs. 0.688 vs. 0.740 vs. 0.757 vs. 0.812. However, it did not outperform the PSAD+MADC model (AUC, 0.949 vs. 0.888 and 0.913 vs. 0.854, respectively) (*P* > 0.05) in the training and testing cohorts, respectively.

## 4 Discussion

Precise definition of PI-RADS 3 lesions in the transition zone (TZ) presents a significant challenge due to the atypical imaging features associated with these lesions. This is complicated by the reality that malignant epithelial cells often associated with csPCa are, in this classification, usually distributed sparsely and arranged around the acinar structures. Subsequently, csPCa imaging and benign conditions like hyperplasia, inflammation, and fibrosis can exhibit significant overlap (19). This can result in near imperceptible changes to MRI signal, leading to high rates of interpretation migration and fair inter-observer agreement (20). Furthermore, prostatic hyperplasia in elderly men, predominantly originating in the TZ, is intrinsically heterogeneous and comprises ill-defined tissues, often mistaken for csPCa due to their cellular and vascular nature (12). Currently, there is a shortage of effective means to refine lesion classification, leaving clinical management unclear.

Radiomic analysis provides a non-invasive tool using existing MRI images to obtain data about target organs and tissues. Its strength lies in eliminating subjective interpretation and observer reliance, concurrently analyzing hundreds of imaging features. This allows for a thorough characterization of tumor heterogeneity, reflecting tumor cellularity, proliferation, angiogenesis, hypoxia, and necrosis (21). Guiding classification, risk stratification, and clinical decision-making measures for suspicious lesions form key aspects of its performance duties (16, 22, 23). Consequently, radiomic analysis shows immense potential in distinguishing csPCas from painless or benign cases (22, 23).

Several studies have probed into the function of radiomics in prostate imaging (24–26). Hou et al. evaluated radiomics machine learning (ML) models and reported an enhanced risk stratification, superior to subjective radiologist evaluation for identifying csPCa in PI-RADS v2 category 3 lesions, with the AUC of radiomics ML models ranging from 0.87–0.89 (24). In a different study, Li et al. utilized a support vector machine (SVM) classification to stratify the Gleason Score (GS) of prostate cancer in the central gland using mpMRI. This approach showcased exceptional performance, with AUC values oscillating between 0.97 (CI 0.94–0.99) and 0.91 (CI 0.85–0.95) (25). Schelb et al. used a U-Net trained with T2-weighted and diffusion-weighted images, thereby achieving a performance on par with that of PI-RADS assessment (26). These studies collectively attest to the superior performance of radiomics in detecting prostate lesions.

Our study exhibits a classification capability that is, at the very least, comparable to those reported in the literature, thereby reiterating the utility of radiomics in prostate MRI. As reported in **Table 4**, the XGboost model showed the best performance, with AUC values of 0.949 and 0.913 in the training and testing cohorts, respectively. This indicates the XGboost model’s robust capacity to recognize csPCa, indicating that machine learning’s potential as an efficient and noninvasive instrument for the prediction of csPCa in PI-RADS 3 lesions. Commonly deployed to address classification issues, XGboost stands as the most accurate model for predicting 1-year survival among non-small cell lung cancer patients diagnosed with bone metastases (27). XGboost can also infer the tissue sources of 10 unique cancer types and outperforms traditional machine learning algorithms (28).

The predictive potential of clinical and radiological biomarkers for diagnosing prostate cancer associated with equivocal PI-RADS 3 lesions undergoing biopsy has been evaluated positively in previous studies (29, 30). For instance, Brancato et al. concluded that the most vital feature for the detection of cancer in PI-RADS 3 lesions was based on ADC maps (31). Our data also supported the use of quantitative ADC measurements for decision-making in PI-RADS 3 lesions, with AUC of 0.856 (0.782–0.923) and 0.788 (0.628–0.920) in the training and testing cohorts, respectively. Efficient at discerning the microenvironment of neoplastic tissues, ADC can identify alterations in compartmental volumes, such as stroma, epithelium, and lumen space, and cellularity (32), It currently serves as best parameter for prostate MRI assessment (4, 33). Moreover, ADC has been consistently proven to be inversely correlated with factors like tumor grade, tumor aggressiveness, and pathological stage (34–36). We compared the performance of the mean ADC with biparametric radiomics to assess whether it had an added value over that of machine learning. The Delong test results revealed superior performance from XGboost models as opposed to the mean ADC model in the training cohorts. However, this superiority was not replicated in the testing cohorts. Moreover, it did not outperform the PSAD+MADC model (AUC, 0.949 vs. 0.888 and 0.913 vs. 0.854, respectively) (P > 0.05) in both the training and testing cohorts. Thus, within the context of our study, ADC values remained the most decisive parameter, aligning with previous studies’ findings (33, 36). Bonekamp et al. (36) compared the performance of biparametric contrast-free radiomics with that of machine learning for detecting csPCa, also concluded that the performance of radiomic machine learning did not exceed that of the mean ADC. This finding is coherent with the results observed in our study.

However, several differences from the present study should be noted. Prior studies did not conduct separate analyses for peripheral zone (PZ) and TZ lesions. Given that the lesion characteristics significantly differ between PZ and TZ, and the primary sequences vary, it is recommended to perform targeted analysis based on lesions in different zones rather than combining them. Second, some studies exploring the intelligent diagnosis of PI-RADS 3 lesions were confined to basic radiomic features (33, 35, 36) and overlooked the additional diagnostic value of clinical indicators. Compared with these similar studies, the present study evaluated clinical features associated with csPCa, encompassing age, tPSA, fPSA, fPSA/tPSA, prostate volume, and PSA density. However, only one of these, specifically PSAD, proved useful for building predictive models.

MRI application as an adjuvant examination rather than a clinical triage tool can pose challenges, considering negative findings do not necessarily discourage further progression to a biopsy, potentially leading to overtreatment (37). Integrating MRI findings with PSAD may mitigate these concerns. PSAD also constitutes an essential component of the best-performing XGboost model in this study, and has been extensively investigated in several studies (38–40). A large multi-institutional collaborative study showed that among the men with a solitary PI-RADS 3 lesion on MRI, nearly 87% of those with a low PSAD had no or only GG1 prostate cancer. In contrast, as PSAD increases, the rate of csPCa detection increases to more than one-third of men biopsied (37). Several studies have identified an independent association of PSAD with csPCa, even in patients with serum PSA levels slightly exceeding or within the normal range–a common occurrence across various clinical scenarios, such as early diagnosis, repeat biopsy, and active surveillance (38). Roscigno et al. (39) reported that higher PSAD was associated with an elevated risk of reclassification, with 0.20 as the threshold in definitive or follow-up biopsy. Washino et al. (40) increased the negative predictive value (NPV) of PI-RADS from 0.84 to 0.96 by using PSAD with a cut-off value of 0.15 ng/mL/cc. Ullrich et al. (41) concluded that if the PSAD cut-off was 0.15 ng/mL^2^, 53% of patients with a PI-RADS v2 score of 3 would have avoided biopsy.

In our study, the predictive performance of radiomics machine learning models did not surpass that of the comprehensive model combining clinical variables and radiological features (MADC + PSAD). This may change with the development of next-generation machine learning techniques for larger-scale cohorts in multicentric setups, as machine learning methods rely on large amounts of training and testing data. These tools typically do not require segmentation or handcrafted radiomic features. In the current study, more traditional machine learning methods were used due to the relatively small sample size and number of csPCa cases.

Our study had several limitations. The retrospective design of this study, combined with the lack of results from radical prostatectomy specimens as a reference standard, means that selection bias and biopsy bias are potential issues. In addition, the PI-RADS 3 dataset is notably small and imbalanced. A more sizable, balanced study group would better facilitate radiomic analyses and aid in formulating robust predictive models. Lastly, identifying PI-RADS 3 lesions can prove challenging, making some lesions ambiguous.

## 5 Conclusions

Radiomics–based algorithms, notably the XGboost models, demonstrated substantial proficiency in predicting csPCa in PI-RADS 3 lesions in TZ. This could potentially elevate the rate of prostate-positive biopsy for PI-RADS 3 while decreasing the incidence of unnecessary biopsies. Predictions yielded by the XGboost classifier could serve as a crucial reference for clinical decision-making. However, in the current cohort, no additional benefits of the radiomic classifiers were observed over the combined model of clinical and radiological findings, suggesting the mean ADC+PSAD as the most generalized and optimal choice for quantitative prostate assessment.

## Data availability statement

The original contributions presented in the study are included in the article/supplementary material. Further inquiries can be directed to the corresponding authors.

## Ethics statement

The studies involving humans were approved by ethics committee of Fuqing City Hospital Affiliated to Fujian Medical University. The studies were conducted in accordance with the local legislation and institutional requirements. The participants provided their written informed consent to participate in this study. Written informed consent was obtained from the individual(s) for the publication of any potentially identifiable images or data included in this article.

## Author contributions

T-HC and Y-SL established the central thrust of our study. Y-YZ and M-LX drafted the manuscript, which was revised by ZX, and L-JD. Y-FL and J-LC analyzed the research data. All authors contributed to the article and approved the submitted version.
